# Linking Foraging Behaviour and Habitat Preferences During Moult Across Multiple Populations of Red‐Throated Diver

**DOI:** 10.1002/ece3.70733

**Published:** 2024-12-23

**Authors:** James Duckworth, Susan O'Brien, Ruth E. Dunn, Ib K. Petersen, Aevar Petersen, Guðmundur Benediktsson, Logan Johnson, Petteri Lehikoinen, David J. Okill, Roni Väisänen, Jim Williams, Stuart Williams, Francis Daunt, Jonathan A. Green

**Affiliations:** ^1^ School of Environmental Sciences University of Liverpool Liverpool UK; ^2^ Scottish Government Marine Laboratory Aberdeen UK; ^3^ Lancaster Environment Centre Lancaster University Lancaster UK; ^4^ The Lyell Centre Heriot‐Watt University Edinburgh UK; ^5^ Department of Bioscience Aarhus University Aarhus Denmark; ^6^ Independent Researcher Reykjavik Iceland; ^7^ Independent Researcher Shetland UK; ^8^ Finnish Museum of Natural History, University of Helsinki Helsinki Finland; ^9^ Avescapes Oy Helsinki Finland; ^10^ Independent Researcher Orkney UK; ^11^ UK Centre for Ecology & Hydrology Midlothian UK

**Keywords:** foraging, *Gavia*, isotope, loon

## Abstract

Understanding the habitat use of individuals can facilitate methods to measure the degree to which populations will be affected by potential stressors. Such insights can be hard to garner for marine species that are inaccessible during phases of their annual cycles. Here, we quantify the link between foraging habitat and behaviour in an aquatic bird of high conservation concern, the red‐throated diver ( *Gavia stellata* ) across three breeding populations (Finland, Iceland and Scotland) during their understudied moult period. Specifically, we quantify the relationship between feather isotope values (δ^13^C and δ^15^N) and diving behaviour, within and between populations, examining the use of intra‐depth zone (IDZ) dives as a proxy for benthic foraging. We found a strong positive relationship between both higher δ^15^N values and, to a lesser extent, δ^13^C values and the proportion of IDZ dives. This relationship was consistent across all three populations, but the baseline δ^13^C values varied between them, indicative of the populations' different moulting areas. Our results demonstrate that red‐throated divers continue to be generalist foragers after their breeding seasons, and that behavioural flexibility varies within and between populations. Furthermore, due to the existence of these relationships, we reveal the potential of stable isotope analysis as a standalone tool for monitoring changes in habitat use in this ecologically significant species. The approach may also apply to other generalist foragers that are known to use multiple foraging strategies (e.g., foraging benthically and pelagically), with implications for future conservation efforts.

## Graphical abstract

We demonstrate a link between foraging habitat and behaviour in an elusive aquatic bird, the red‐throated diver ( *Gavia stellata* ) across three populations (Finland, Iceland and Scotland) during their understudied moult period. We found strong positive relationships between feather isotope signatures (δ^13^C and δ^15^N) and the proportion of benthic rather than pelagic dives in all three populations. Our results demonstrate that red‐throated divers continue to be generalist foragers after their breeding seasons, and that behavioural flexibility varies within and between populations.

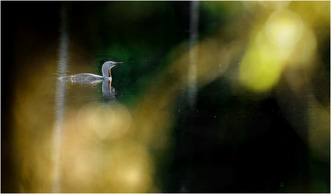

## 1 Introduction

Insights into a species' habitat use, and how this varies within and between populations can facilitate its monitoring and conservation by allowing us to more explicitly link individuals to threats (Bro‐Jørgensen, Franks, and Meise 2019). For predators, information on prey preferences and fine‐scale foraging behaviour can provide insights into their foraging habitats (Bailleul et al. 2010). For example, knowledge of the isotopic composition of predator tissue, inferred via stable isotope analysis, can provide information on where it feeds and what on (Peterson and Fry 1987; Chiaradia et al. 2014). Specifically, variation in specific nitrogen isotopes (δ^15^N) can reveal the trophic level that an organism feeds at, whereas variation in specific carbon isotopes (δ^13^C) reflect the primary sources of carbon within a food web (Peterson and Fry 1987). Furthermore, fine‐scale biologging data can reveal variation in habitat use and foraging behaviour over space and time, such as GPS logger data revealing that black‐legged kittiwake ( *Rissa tridactyla* ) foraging habitat selection changed over the tidal cycle (Trevail et al. 2018). In this way, knowledge of both predator stable isotope composition and foraging behaviour from biologging can yield insights into how foraging and habitat use varies both within and between populations (Ito et al. 2009). Inferring foraging behaviour and habitat use and the resultant risks of potential threats is particularly important for species and/or periods of the annual cycle when individuals are inaccessible and difficult to study.

Although during their breeding seasons many aquatic birds are central place foragers (Orians and Pearson 1979), they typically migrate upon release from the constraints of breeding, with different species, populations and individuals displaying a diversity of migratory strategies that can make them less accessible for study (Phillips et al. 2017; Buckingham et al. 2022). Furthermore, the challenges of the non‐breeding season often include demanding conditions and events that influence foraging behaviour, such as feather moult which is an essential yet intrinsically energetically costly process during which some species are flightless (Dunn et al. 2019). As a result of their migratory ecology, understanding of aquatic bird foraging ecology and habitat preferences during the non‐breeding season are therefore generally less well developed, despite it being a period of high mortality (Harris, Frederiksen, and Wanless 2007). This knowledge gap can in part be solved by applying approaches developed during the breeding season to the increasing number of datasets being obtained on non‐breeding foraging behaviour. For example, during the breeding season, the study of intra‐depth zone (IDZ) dives has been proposed as a metric to infer whether diving predators target benthic or pelagic habitats and prey (Tremblay and Cherel 2000; Knox, Baylis, and Arnould 2017; Tessier and Bost 2020). This measure gives an estimate of whether a given bout has repeated dives to a similar depth, indicating foraging is more likely to be benthic (foraging on the sea floor) or has repeated dives to varying depths, indicating foraging is pelagic (foraging in the water column), based on a higher or lower proportion of IDZ dives, respectively (Tremblay and Cherel 2000). Use of this metric has been less common during post‐breeding (Buckingham et al. 2023), and is not commonly validated for any time period. Red‐throated divers ( *Gavia stellata* ) are an understudied aquatic bird that, like other divers, forages with a mix of benthic and pelagic dives (Duckworth et al. 2021; Kenow et al. 2023). Red‐throated divers make proportionally fewer IDZ dives over the course of their breeding season (Duckworth et al. 2021), likely reflective of their generalist foraging strategy allowing them to switch between shallowly diving within freshwater and marine ecosystems, as well as between benthic and pelagic habitats (Duckworth et al. 2020, 2021). Even less is known about this species in the non‐breeding season, though previous stable isotope analysis has revealed that Baltic Sea red‐throated divers had a largely pelagic diet (ca. 50% of prey) during the winter (Morkūnė et al. 2016). These two quantities, that can indicate individual and population variation in habitat use, have rarely been compared to each other in any species (Harris et al. 2016). Validating the relationship between foraging habitat use inferred from stable isotope analysis and IDZ dives in red‐throated divers during post‐breeding would enable greater insights into the foraging behaviour of this species at this crucial time of year.

Red‐throated divers are susceptible to anthropogenic threats such as disturbance from shipping and offshore wind farms (Mendel et al. 2019; Heinänen et al. 2020; Garthe et al. 2023) and so there is an increasing interest in their habitat use and foraging behaviour (Dunn et al. 2024). The lack of knowledge regarding red‐throated diver post‐breeding foraging ecology is particularly concerning due to this likely being a time when they experience increased exposure to risks (Dierschke et al. 2017). Indeed, shortly after their breeding season, red‐throated divers moult their flight feathers and become flightless for a period of approximately 3 weeks, being unable to move long distances and avoid threats during this time (Kleinschmidt et al. 2022). This feather moult period is one of the most energetically demanding of the avian annual cycle (Newton 2011) and a closely related species, the great northern diver ( *Gavia immer* ), experiences episodic die offs associated with this period, during which dead birds appear to be emaciated (Forrester et al. 1997). Different species and populations are likely to experience varying environmental conditions and potential threats, and so more in‐depth, multifaceted studies of diver prey preferences, foraging behaviour and habitat use are needed to understand how their ecology might vary between populations during post‐breeding (Paruk et al. 2021; Duckworth et al. 2022; Mager et al. 2022). Establishing a baseline link between foraging behaviour and stable isotope values can contribute to this understanding, as stable isotope values in feathers may prove to be valuable, low‐impact, early warning indicators of changes in habitat use or location in this species (Hobson 2007).

In this study, we used biologging technology at sites in Finland, Iceland and Scotland to determine the foraging behaviour of red‐throated divers from multiple populations during the moult period of their annual cycle. We examined IDZ dives, to determine the degree to which birds targeted benthic (and demersal) or pelagic habitats. Simultaneously, the isotope values of the same individuals were determined with bulk stable isotope analysis of feathers grown during the moult period, to provide a further indication of habitat use during this time. We then quantified the relationship between stable isotopes and foraging behaviour. This enabled us to describe both individual and population‐level habitat use, for a species and phase of the annual cycle that is extremely understudied. We discuss the potential use of stable isotope analysis alone to monitor changes in habitat use for this species of high conservation concern in the future.

## 2 Materials and Methods

### 2.1 Data Collection

Breeding adult birds were caught at their breeding sites in southern Finland, north‐eastern Iceland and the Scottish archipelagos of Orkney and Shetland (see Duckworth et al. (2021) for a map of tagging sites). Bird were captured using a mixture of suspended mist nets and nest traps (*n* = 32, 19 and 38 individual birds caught, respectively) during the 2018 and 2019 breeding seasons (Thompson et al. 2022). Time‐depth recorders (TDR) and light‐based geolocators were deployed on each captured bird (combined weight of loggers was < 1% body mass, in all cases). Tagging locations are shown in Duckworth et al. (2021), moult locations (the Baltic Sea for Finnish birds, the North coast of Iceland for Icelandic birds and around the northern Scottish Isles for Scottish birds) are shown in Duckworth et al. (2022). Upon recovery of the archival tags in 2019 and 2020, one secondary flight feather and one secondary covert feather clipping were taken per individual, from the distal 2 cm of the feather. The collected feathers were assumed to have grown during the moult period. We could not exactly identify when moult occurred with the data we had available, so assumed a common moult period when the birds were highly likely to have spent most of their time in moult (10 September–10 October; Duckworth et al. 2022). Secondary feathers are thought to be less invasive to sample than primary flight feathers, with coverts potentially even less invasive than secondaries (Duckworth et al. 2022). Thus, sampling both secondary flight and covert feathers allowed us to investigate the extent to which average isotopic values varied between feather type during the moult period and make recommendations for future studies. Of the 26 individuals that were recaptured, 11, 9 and 6 individuals from Finland, Iceland and Scotland, respectively, had both a functional TDR and at least one successfully analysed feather sample (Thompson et al. 2022).

### 2.2 Stable Isotope Analysis

Feathers were stored in paper envelopes at room temperature for 4 months prior to isotope analysis, which was carried out by Elemtex Ltd. (Cornwall, UK). Samples were washed three times in a solution of 2:1 v/v chloroform and methanol and rinsed in distilled water to remove external lipids, before being oven‐dried at 60°C. Samples were then weighed into tin capsules using a microbalance and were subsequently run on an ANCA/2020 isotope ratio mass spectrometer, which was set to run in continuous flow mode. Finally, data were normalised to Vienna PeeDee Belemnite for δ^13^C and N2 Air for δ^15^N using USGS40 and USGS41A as reference materials, with typical precisions (standard deviation of standards) being better than 0.3‰. Isotope values are expressed as δ^15^N and δ^13^C, which represents the difference, in parts per thousand, of the ^15^N/^14^N and ^13^C/^12^C isotopes, relative to their respective standard.

### 2.3 Processing Dive Data

TDRs were programmed to record pressure data every 6 s on every fifth day after deployment, to conserve battery life. The collected feathers were assumed to provide a snapshot of the environment and food sources of divers during this time, and so we only analysed TDR data from the moult period (see above; 10 September to 10 October). While the chosen period may not exactly reflect the full moult period, due to uncertainty regarding the exact timings of diver feather moult across the geographical range of the species (Kleinschmidt et al. 2022), using a shorter time frame risked missing individuals that were moulting earlier or later. This therefore ensured that no foraging behaviour outside of the moult period was inadvertently included in analysis and erroneously compared with isotope values of feathers grown at a different time.

TDR tags were processed and dive events, defined as any recorded depth below 1 m, were extracted (Duckworth et al. 2021). These dives were then clustered into foraging bouts, where each bout was classified as at least three related dives with less than 66 s between each dive (Duckworth et al. 2022). In a study of great northern divers, high resolution location data enabled the matching of dive locations to local bathymetry (Kenow et al. 2023). Estimates of location from our geolocator tags were far inferior to this, and as a result, it was not reasonable to attempt to estimate local seafloor depth and accurately classify benthic dives. Instead, we used the occurrence of IDZ dives as a proxy for benthic diving. The number of IDZ dives was defined as the number of dives where the maximum dive depth fell within 90% of the maximum dive depth of the preceding dive within a foraging bout (Tremblay and Cherel 2000). For analysis, an individual's ‘proportion of IDZ dives’ was calculated as the number of IDZ dives divided by the total number of dives in bouts (minus the number of bouts since the first dive in a bout cannot be assessed for being an IDZ dive) and encompassed the entire moult period.

### 2.4 Statistical Analyses

Linear mixed effect regression models were created for two response variables (δ^15^N and δ^13^C) to assess the effect of population, proportion of IDZ dives and feather type. This was to investigate the extent to which average isotopic values varied among populations (Finland, Iceland and Scotland), with pelagic versus benthic foraging (proportion of IDZ dives) and with the feather type (secondary flight feather vs. secondary covert feather) from which samples were obtained. While the sex of most individuals was known with reasonable confidence from field observations and measurements, we felt that sample sizes were too small to consider in our analyses. The full model included the following proportion of IDZ dives, population, feather type, an interaction between feather type and proportion of IDZ dives and an interaction between population and proportion of IDZ dives. All models included the ID of the individual as a random effect to account for some individuals being tagged and sampled in 2 years (Zuur et al. 2009). All nested versions of this model were also generated, with all combinations of fixed effects (number of model combinations = 13), and corrected delta Akaike Information Criterion (∆AICc) values were used to assess the ability of each model to explain the variance within data, compared to the best fitting model (Bozdogan 1987). Model averaging was carried out on all models within four ∆AICc units of the best fitting model. Using four ∆AICc units means we include models with less empirical support; therefore inferences from the averaged model focus on effect size. For each term, the effect size reflects both the weight of the model it occurs in and the effect size it has within that model (Zuur et al. 2009). The averaged model for each of the response variables is referred to here as the ‘final model’, with the weight of each candidate model in the final model being based on their AICc value. We checked that the final models met the assumptions of normality and homogeneity of variances by inspecting Q–Q plots and plots of the residuals versus fitted values. The importance of each variable and interaction is discussed by considering (1) its effect size in the final model, (2) the prevalence in the top (∆AICc < 4) models and final model and (3) the overall support for the models in which they occur. All analyses were done in R version 4.2.1. (R Core Team 2022).

## 3 Results

We obtained data on the diving behaviour and isotopic values of 26 red‐throated divers during their moult period. During their deployment and recording periods, we obtained information on between 376 and 2379 dives (mean = 831 ± 427 SD) for birds that bred in Finland, between 853 and 2849 dives (mean = 1871 ± 631 SD) for birds that bred in Iceland, and between 684 and 1630 dives (mean = 1070 ± 343 SD) for birds that bred in Scotland. Of these dives, a mean of 45% (SD = 17%) were IDZ dives, that is, those where the maximum dive depth fell within 90% of the maximum dive depth of the preceding dive. Values of δ^15^N and δ^13^C were obtained from the secondary flight feathers and secondary covert feathers of all individuals. Within the secondary flight feathers, nitrogen stable isotope values ranged from 12.29‰ to 18.97‰ δ^15^N (mean = 15.45 ± 1.61‰ SD), and values of the secondary coverts ranged from 12.75‰ to 18.99‰ δ^15^N (mean = 15.56 ± 1.65‰ SD). Regarding carbon stable isotopes, secondary flight feather values ranged from −22.58‰ to −16.36‰ δ^13^C (mean = −19.31 ± 2.10‰ SD), and secondary covert values ranged from −22.41‰ to −17.04‰ δ^13^C (mean = −18.93 ± 1.76‰ SD).

**FIGURE 1:**
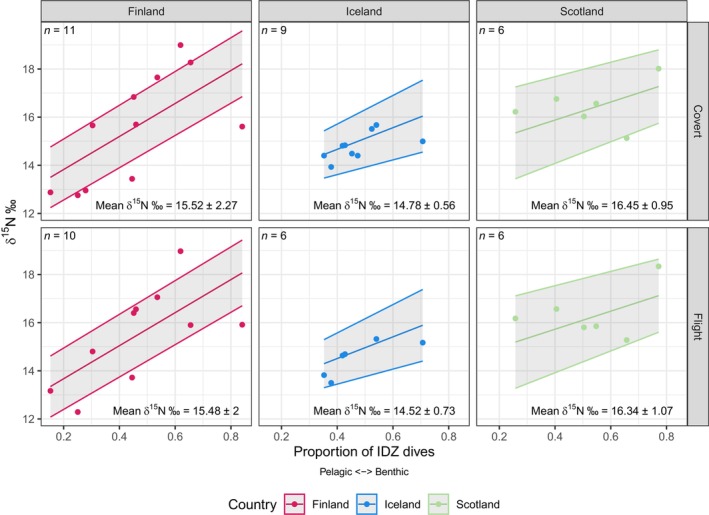
δ^15^N values across the three populations and two feather types sampled in this study against the proportion of IDZ dives taken across the predicted moult period generated by the final averaged model. Populations are represented by red, blue and green for Finland, Iceland and Scotland, respectively. Shaded regions show standard error of the model. Sample sizes are shown in the top left and means and standard deviations of δ^15^N values are shown in the bottom left.

The relative difference in the explanatory power of the δ^15^N models was small, evidenced by the high number of models within 4 AICc units of the best fitting model (Table 1). However, the proportion of IDZ dives appeared in all candidate models (Table 1), suggesting this was an important predictor for δ^15^N. This was reflected in the final averaged model including a strong positive relationship between the proportion of IDZ dives and δ^15^N (Figure 1). Values of δ^15^N were predicted to change by 6.86‰, 4.50‰ and 3.74‰ across Finland, Iceland and Scotland, respectively, when going from an IDZ of 0 to 1 (i.e., from pelagic to benthic dives). Feather type also appeared in the top ranked δ^15^N model, plus several of the other candidate models; however, the effect size of feather type in the final averaged model was small (causing a difference of −0.15‰; Table 2) and had a weak interaction with the proportion of IDZ dives. For instance, the predicted δ^15^N values for the flight and covert feathers in Finland for an individual with a predicted 0.84 proportion of IDZ dives in the final model was 18.1‰ and 18.2‰, respectively. Population had a strong effect on δ^15^N and appeared in many of the candidate models, along with an interaction with the proportion of IDZ dives. The largest difference was between Finland and Scotland, where the gradient of the slope between δ^15^N and proportion of IDZ dives was 6.9‰ and 3.7‰, respectively.

**TABLE 1:** Top models ranked by ∆AICc values where ∆AICc values were < 4.

Models	∆AICc	Degrees of freedom	Weight
**δ** ^**15**^ **N models**			
δ^15^N ~ IDZ + feather	0 (AICc = 135.4)	5	0.202
δ^15^N ~ IDZ	0.07	4	0.196
δ^15^N ~ IDZ + population	0.78	6	0.137
δ^15^N ~ IDZ + population + population:IDZ	0.87	8	0.131
δ^15^N ~ IDZ + population + feather	0.92	7	0.128
δ^15^N ~ IDZ + population + feather + population:IDZ	1.01	9	0.122
δ^15^N ~ IDZ + feather + feather:IDZ	2.61	6	0.055
δ^15^N ~ IDZ + population + feather + feather:IDZ	3.81	8	0.03
**δ** ^**13**^ **C models**			
δ^13^C ~ population + IDZ	0 (AICc = 123.8)	6	0.673
δ^13^C ~ population + IDZ + feather	2.35	7	0.208
δ^13^C ~ population + IDZ + population:IDZ	3.47	8	0.119

*Note:* The ∆AICc column shows the number of AICc units away from the best fitting model that each model was. ‘IDZ’ represents the proportion of intra‐depth zone dives, ‘feather’ represents feather type (secondaries or coverts) and interactions are denoted by colons between variables.

**TABLE 2:** Final averaged model for Nitrogen and Carbon models.

Response	Intercept	Population			Feather		IDZ			IDZ:feather	
		Finland	Iceland	Scotland	Covert	Flight	Finland	Iceland	Scotland	Covert	Flight
δ^15^N	12.45	~	0.41	1.93	~	−0.15	6.86	4.5	3.74	~	0.005
δ^13^C	−22.68	~	2.73	3.96	~	0.03	3.24	3.08	2.56	~	~

*Note:* The intercept is set for population = Finland, feather = Covert, IDZ = 0. A tilde (~) indicates no difference from the intercept. Population shows the effect of population on the intercept. Feather shows the effect of feather type on the intercept. IDZ represents the increase in the response variable from the intercept for an increase in the proportion of IDZ dives from 0 to 1, with each population representing the population‐specific effect of the proportion of IDZ dives after accounting for an interaction. ‘IDZ: Feather’ represents the interactive effect of feather type on the relationship between IDZ and isotope value, per unit increase of proportion of IDZ dives.

**FIGURE 2:**
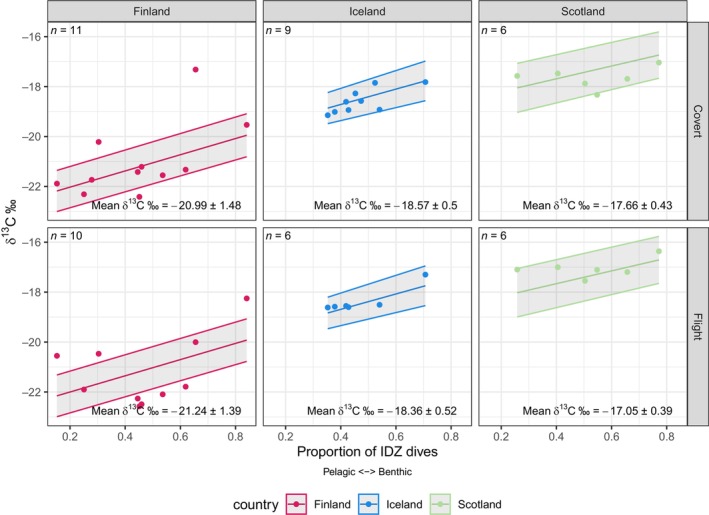
δ^13^C values across the three populations and two feather types sampled in this study against the proportion of IDZ dives taken across the predicted moult period generated by the final averaged model. Populations are represented by red, blue and green for Finland, Iceland and Scotland, respectively. Shaded regions show standard error of the model. Sample sizes are shown in the top left and means and standard deviations of δ^15^N values are shown in the bottom left.

The final averaged model for δ^13^C showed a much larger effect of population (Table 2 and Figure 2), with it being included in all candidate models (Table 1). The largest difference in δ^13^C was between Finland and Scotland, with an estimated difference of 3.96‰ (SE 0.49) δ^13^C in the final model (Figure 2). Values of δ^13^C were weakly affected by the proportion of IDZ dives, with ‘IDZ’ appearing in all top models, but having a much weaker effect size than it did on δ^15^N (estimated 3.2‰ δ^13^C change from a value of 0 to 1 for the proportion of IDZ dives). While an interaction between the proportion of IDZ dives and population was in the final model for δ^13^C (Table 1), the effect size was weak, with a maximum difference of 0.68‰ between the slope of the Finland and Scotland populations (Figure 2). Only one candidate model included feather type, with an effect size of 0.02‰, indicating that there was no strong difference in δ^13^C between secondary flight and secondary coverts when comparing between populations and foraging behaviour.

## 4 Discussion

We have shown that, following their breeding seasons, red‐throated divers engage in a mix of pelagic and benthic foraging. Values of δ^15^N and δ^13^C varied both within and between populations and were both positively correlated with the proportion of IDZ dives. Despite carbon isotopes often being thought of as indicative of foraging habitat and nitrogen isotopes being indicative of foraging trophic level (Peterson and Fry 1987), proportions of IDZ dives, a proxy for benthic diving, were most strongly correlated with δ^15^N values (Table 2). This result is corroborated by benthic prey species (e.g., European smelt *Osmerus eperlanus* ) in the moulting areas of red‐throated divers having higher δ^15^N values than shoaling prey species (e.g., Baltic herring *Clupea harengus* and Baltic sprat *Sprattus sprattus* , two of the most dominant pelagic fishes in the North Atlantic; Nfon, Cousins, and Broman 2008; Morkūnė et al. 2016). Benthic fishes having comparatively high δ^15^N is likely due to them feeding among and having close associations with benthos which is enriched in ^15^N (Nfon, Cousins, and Broman 2008). Indeed, the differences in δ^15^N values between benthic and pelagic prey species (ca. 4‰; Nfon, Cousins, and Broman 2008; Morkūnė et al. 2016) align with the range of δ^15^N values observed in the Finnish population of red‐throated divers in this study (Figure 1). Our results therefore add weight to the assumption that IDZ diving is indicative of red‐throated divers foraging benthically, with birds serially diving to similar depths in order to exploit benthic habitats (Duckworth et al. 2021), even during post‐breeding.

Variation in values of δ^13^C between red‐throated diver populations, is likely due to spatial variation in baseline δ^13^C values (St. John Glew et al. 2019). Indeed, previous work on red‐throated divers from Finland, Iceland and Scotland revealed that individuals from each population likely moult in discrete geographical areas, namely the Baltic Sea, the North coast of Iceland, and around the northern Scottish Isles, respectively (Duckworth et al. 2022; Kleinschmidt et al. 2022). Therefore, while inter‐population variation in δ^13^C values is likely reflective of population‐differences in moulting areas (Bearhop et al. 2006), intra‐population differences are instead likely to be driven by individual differences in foraging ecology. As δ^13^C values are generally higher in benthic food webs than in pelagic food webs, the positive relationship that we observed between δ^13^C and the proportion of IDZ dives (Figure 2), although less strong than that between δ^15^N and IDZ diving, offers more support of IDZ dives being indicative of a benthic foraging strategy in post‐breeding red‐throated divers.

Populations of red‐throated divers from Iceland, Scotland and Finland demonstrated differences in the extent of their IDZ diving. Individual variation in IDZ diving was most prominent in birds from Finland (Figures 1 and 2), indicative of individuals adopting particular foraging strategies and acting as specialists in a generalist population (Vander Zanden et al. 2010). Scrutiny of individual‐level data (Figure S1) confirms that individual birds from Finland were more likely to dive either benthically or pelagically in a large proportion of their bouts. Previous work in the Baltic Sea inferred that wintering red‐throated divers had largely pelagic diets (Morkūnė et al. 2016), but our results suggest that benthic foraging can constitute a significant proportion of the diet of some individuals during their moult. Conversely, the range in the proportion of IDZ dives performed by the population of red‐throated divers from Scotland and especially Iceland was smaller than birds from Finland. Scottish and Icelandic birds had more bouts with intermediate IDZ values, suggesting more intra‐individual variability in foraging strategy within foraging bouts (Figures S2 and S3). Overall, the variation that we observe reveals that similar to their breeding seasons (Duckworth et al. 2021), red‐throated divers continue to be generalist foragers when they depart their breeding areas, though the degree of variation between individuals can be different between populations.

The prevalence of the proportion of IDZ dives as an explanatory variable in the models for both δ^15^N and δ^13^C, lends strong support for feather samples alone to be able to indicate foraging behaviour in red‐throated divers. Furthermore, there was little evidence that the effects of population and foraging behaviour on isotope values differed between feather types (i.e., secondary flight feathers and secondary covert feathers), indicating that the two feather types were grown simultaneously, using the same food source. This corroborates previous work illustrating that both secondary flight feathers and secondary covert feathers have relatively even power in predicting red‐throated diver moult locations (Duckworth et al. 2022). Our results now also suggest that among generalist foragers, where individuals vary in their habitat use and exhibit a range of foraging strategies, a single feather sample, grown during the period of interest, may be enough to reveal new insights into the foraging ecology of both individuals and populations. In the case of red‐throated divers, δ^15^N has more potential in this regard, due to its stronger relationship with the proportion of IDZ dives (in comparison with that of δ^13^C; Figure 1). Inferring foraging behaviour and habitat by taking feather samples from breeding birds and analysing them for nitrogen isotopes is likely to have less of an impact on individuals than biologging studies and does not require the recapture of individuals (Gillies et al. 2020). Furthermore, in the context of marine birds that undergo seasonal synchronous moults of their flight feathers, only secondary covert feathers should be sampled, as they play a lesser role in flight and so their loss has lower associated costs (Rohwer and Rohwer 2013). We therefore demonstrate that, following initial work to understand how isotopic values vary between foraging behaviours and habitats, using feathers samples has benefits to increase our understanding of the frequency of differing foraging strategies. This minimally invasive technique could therefore be particularly valuable when investigating populations that are at risk of decline, such as those where there is concern that anthropogenic activity may deter individuals from their foraging habitats (Heinänen et al. 2020). In addition to being a minimally invasive way of investigating such populations, feather sampling for stable isotope analysis could also have applications for species conservation and protection, particularly where threats may vary depending on habitat use. Indeed, previous work on northern gannets ( *Morus bassanus* ) has linked isotope values to the importance of fisheries discards in the diets of individuals, with implications for their conservation (Votier et al. 2010). In the case of red‐throated divers, by estimating the proportion of individuals that use different foraging habitats, the quantification of the detrimental effects of habitat removal or degradation on a population is enabled. This use of stable isotope analysis in determining habitat use might also be important for red‐throated divers caught at sea (e.g., Kleinschmidt et al. 2022). By catching individuals during the winter, near or within areas of offshore wind development and other anthropogenic activity, insights might be garnered into whether those individuals fed benthically or pelagically during their moult and how this might change between years. Future work might also consider whether adding analysis of sulphur isotopes and/or putative prey species isotope analysis might give further insights into non‐breeding behaviour (Morkūnė et al. 2016).

In conclusion, we identified a relationship between a foraging metric (the proportion of IDZ dives) and the stable isotope values of moulting red‐throated divers from multiple populations. Our results indicate that intra‐population variation in habitat use and foraging strategy can be quantified with isotope values, though the relationship and baseline isotope values for the population should be determined first. We propose that beyond just information on diet, stable isotope values can provide a less invasive way to gain insight into the behaviour of other generalist foraging aquatic bird species. This finding may be especially for populations, species and times of year that are hard to study. In this way, we might be able to understand change in aquatic bird foraging ecology and habitat use and better apportion the effects of habitat‐specific stress.

## Author Contributions

**James Alexander Duckworth:** conceptualization (equal), formal analysis (lead), investigation (equal), methodology (lead), visualization (supporting), writing – original draft (supporting), writing – review and editing (supporting). **Susan O'Brien:** conceptualization (equal), funding acquisition (lead), investigation (equal), methodology (equal), project administration (equal), resources (equal), supervision (supporting), writing – review and editing (equal). **Ruth E. Dunn:** data curation (supporting), formal analysis (supporting), visualization (supporting), writing – original draft (lead), writing – review and editing (lead). **Ib Krag Petersen:** conceptualization (equal), funding acquisition (equal), investigation (equal), methodology (equal), project administration (equal), supervision (supporting), writing – review and editing (equal). **Aevar Petersen:** conceptualization (equal), data curation (equal), funding acquisition (equal), investigation (equal), methodology (equal), project administration (equal), writing – review and editing (equal). **Guðmundur Benediktsson:** methodology (equal), writing – review and editing (equal). **Logan Johnson:** methodology (equal), writing – review and editing (equal). **Petteri Lehikoinen:** methodology (equal), writing – review and editing (equal). **David J. Okill:** methodology (equal), writing – review and editing (equal). **Roni Väisänen:** methodology (equal), writing – review and editing (equal). **Jim Williams:** methodology (equal), writing – review and editing (equal). **Stuart Williams:** methodology (equal), writing – review and editing (equal). **Francis Daunt:** conceptualization (equal), methodology (equal), supervision (supporting), writing – review and editing (equal). **Jonathan A. Green:** conceptualization (equal), funding acquisition (equal), investigation (equal), methodology (equal), project administration (equal), supervision (lead), writing – original draft (equal), writing – review and editing (equal).

## Conflicts of Interest

The authors declare no conflicts of interest.

## Supporting information


Appendix S1.

## Data Availability

Raw data and the R‐script used for analysis are included in the Supporting Information.
