# Supplementary material for: Linking Foraging Behaviour and Habitat Preferences During Moult Across Multiple Populations of Red‐Throated Diver
Source: Ecol Evol. 2024 Dec 23;14(12):e70733. doi: 10.1002/ece3.70733 (PMC11664317; doi:10.1002/ece3.70733)
Supplement: Supplementary file 1 — Appendix S1. [file ECE3-14-e70733-s001.zip › Supplmentary_figures.docx]

**Linking non-breeding foraging behaviour and habitat preferences across multiple populations of red-throated diver**

Supplementary Figures

Authors

Duckworth J.^1^, O’Brien S.^2,3*^, Dunn, R.E.^4,5^, Petersen I.K.^6^, Petersen A.^7^, Benediktsson G.^8^, Johnson L. ^9^, Lehikoinen P.^10,11^, Okill D. J.^8^, Väisänen R.^10^, Williams J. ^8^, Williams S. ^8^, Daunt F.^11^, Green J. A.^1^

Author affiliations

^1^ School of Environmental Sciences, University of Liverpool, Brownlow Street, Liverpool, L69 3GP, UK;

^2^ Joint Nature Conservation Committee, Inverdee House, Baxter Street, Aberdeen, AB11 9QA, UK;

^3^ Marine Scotland Science, Marine Laboratory, PO Box 101, 375 Victoria Road, Aberdeen AB11 9DB, UK;

^4^ The Lyell Centre, Heriot-Watt University, Edinburgh, EH14 4BA, UK;

^5^ Lancaster Environment Centre, Lancaster University, Lancaster, LA1 4YQ, UK;

^6^ Department of Bioscience, Aarhus University, Denmark;

^7^ Independent researcher, Brautarland 2, 108 Reykjavik, Iceland;

^8^ Independent researcher;

^9^ Finnish Museum of Natural History, P. Rautatiekatu 13, 00014 University of Helsinki, Finland;

^10^ Avescapes Oy, Vihdintie 3-5 B 21, 00320 Helsinki, Finland;

^11^ UK Centre for Ecology & Hydrology, Bush Estate, Penicuik, Midlothian, EH26 0QB, UK;

*Present address: MacArthur Green, 93 South Woodside Road, Glasgow, G20 6NT, UK.

Corresponding Author: Jonathan Green, Jonathan.Green@liverpool.ac.uk


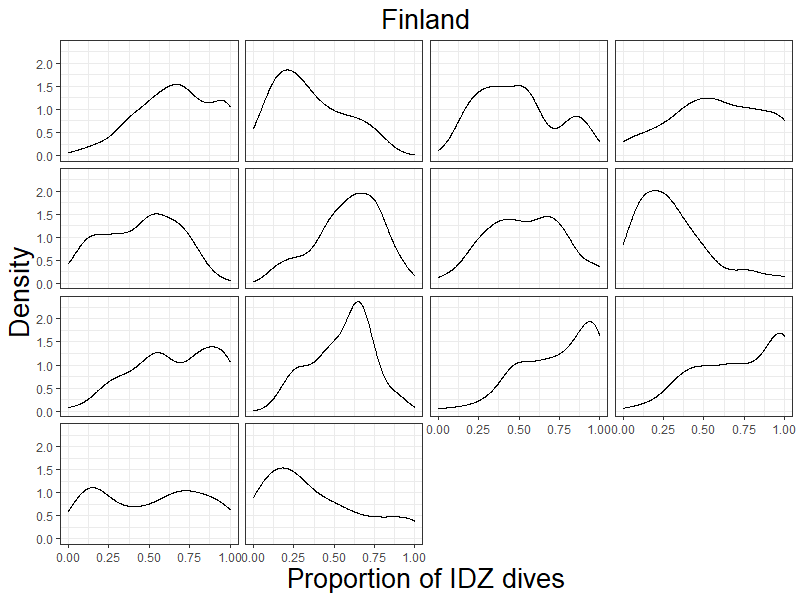


Supplementary Figure 1: A density histogram showing the proportion of intra-depth zone (IDZ) dives in each bout, where the number of dives was at least eight for the Finland population of red-throated divers. The bandwidth is the standard deviation of the smoothing kernel for each panel, which itself is generated from a Gaussian distribution. Bouts are not weighted for the number of dives within them.


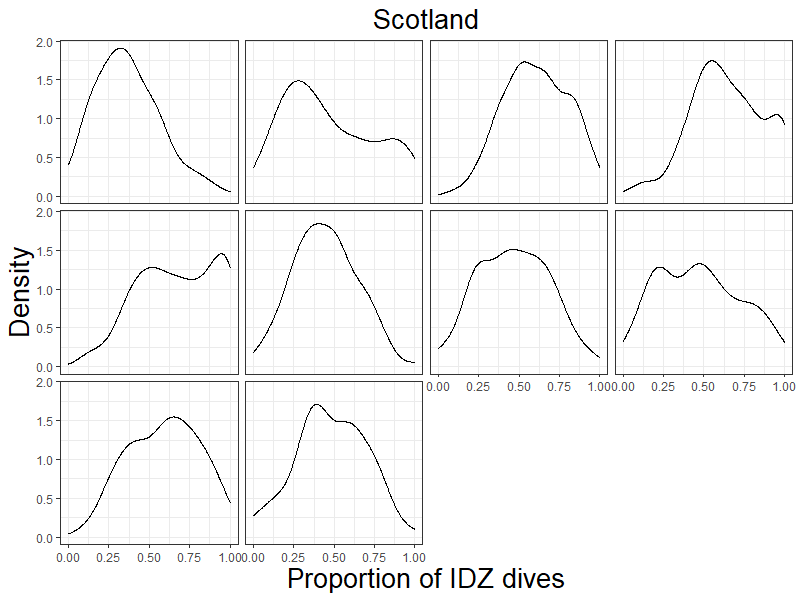


Supplementary Figure 2: A density histogram showing the proportion of intra-depth zone (IDZ) dives in each bout, where the number of dives was at least eight for the Scotland population of red-throated divers. The bandwidth is the standard deviation of the smoothing kernel for each panel, which itself is generated from a Gaussian distribution. Bouts are not weighted for the number of dives within them.


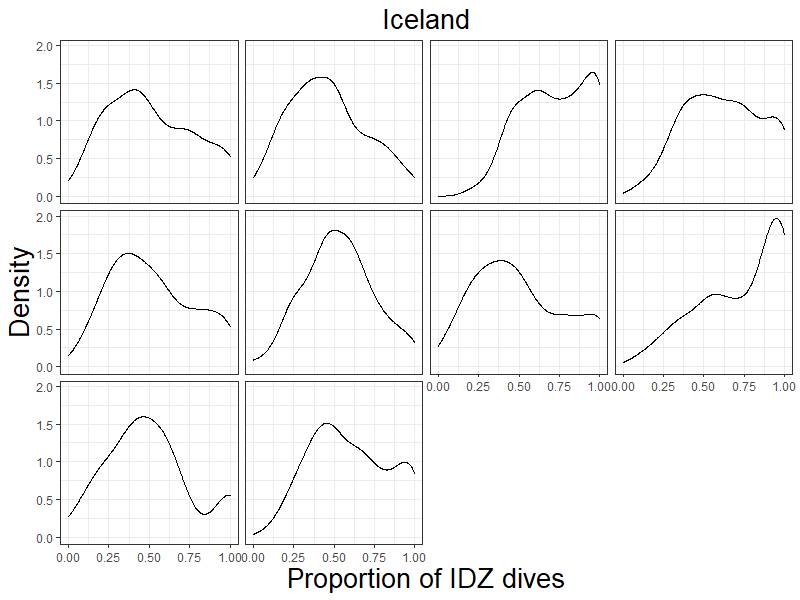


Supplementary Figure 3: A density histogram showing the proportion of intra-depth zone (IDZ) dives in each bout, where the number of dives was at least eight for the Iceland population of red-throated divers. The bandwidth is the standard deviation of the smoothing kernel for each panel, which itself is generated from a Gaussian distribution. Bouts are not weighted for the number of dives within them.
